# Data Sharing Practices & Experiences Among AD/ADRD Investigators with International Collaborations

**DOI:** 10.1002/alz70856_104322

**Published:** 2025-12-26

**Authors:** Catherine E. A. Scipion, Karen E. Nielsen, Jalayne J. Arias

**Affiliations:** ^1^ School of Public Health ‐ Georgia State University, Atlanta, GA, USA

## Abstract

**Background:**

Data sharing within international collaborations enhances research by expanding dataset diversity, improving reproducibility, and fostering cross‐border knowledge exchange‐ leading to stronger evidence and scientific discoveries. However, little is known about AD/ADRD investigators with international collaborations and their data‐sharing practices and experiences. Understanding these patterns is essential for optimizing global research and ensuring equitable participation. This study characterizes AD/ADRD researchers working with and without international collaborators and examines their data‐sharing practices and experiences.

**Method:**

A NIH‐funded survey (NIH‐1R01AG080093‐01) collected self‐reported cross‐sectional data on data‐sharing practices and experiences. This analysis examined whether working with international collaborators influenced data‐sharing practices or experiences. Demographics, data‐sharing practices, and experiences were compared between groups using Chi‐squared tests (α < 0.05), with Benjamini‐Hochberg correction for multiple comparisons.

**Result:**

Of the 585 survey respondents, 193 (33%) worked with international collaborators. Gender [χ^2^(2) = 9.27, *p* =  0.009] and race/ethnicity [χ^2^(3) = 13.24, *p* =  0.004] distributions significantly differed, with men (60.6% vs. 50.3%) and White researchers (73.6% vs. 61.1%) more represented among those working with international collaborators. Researchers with international collaborations had longer research experience [χ^2^(3) = 35.99, *p* < 0.001], with more having conducted ADRD research for 20+ years (23.3% vs. 10.9%). They were more often ADRC investigators (38.9% vs. 26.4%) [χ^2^(1) = 8.79, *p* =  0.003] and more likely to analyze/generate data outside the U.S. (25.9% vs. [χ^2^(1) = 94.71, *p* < 0.001]. Additionally, they had higher publication rates, with 26.9% publishing 11+ articles vs. 10.1% [χ^2^(2) = 36.95, *p* < 0.001]. They were also more likely to make (64.1% vs. 38.6%) [χ^2^(1) = 32.35, *p* < 0.001] and receive (59.1% vs. 37.0%) [χ^2^(1) = 24.39, *p* < 0.001] formal data‐sharing requests. However, researchers with international collaborators were more likely to report negative data‐sharing outcomes (43.8% vs. 20.3%) [χ^2^(1) = 14.88, *p* < 0.001]. All *p*‐values remained significant after multiple comparison correction.

**Conclusion:**

These findings highlight the role of international collaborations in advancing AD/ADRD research through data sharing, while revealing disparities in researcher representation. Future studies should examine mechanisms linking international collaboration with data‐sharing practices and their broader impact on AD/ADRD research equity.

